# Effects of supplementing coated methionine in a high plant-protein diet on growth, antioxidant capacity, digestive enzymes activity and expression of TOR signaling pathway associated genes in gibel carp, *Carassius auratus* gibelio

**DOI:** 10.3389/fimmu.2024.1319698

**Published:** 2024-04-05

**Authors:** Yingying Du, Xiaowen Lin, Xianping Shao, Jianhua Zhao, Hong Xu, Clement R. de Cruz, Qiyou Xu

**Affiliations:** ^1^ College of Life Science, Huzhou University, Huzhou, China; ^2^ Zhejiang Provincial Key Laboratory of Aquatic Bioresource Conservation and Development Technology, Huzhou University, Huzhou, China; ^3^ Nation Local Joint Engineering Laboratory of Aquatic Animal Genetic Breeding and Nutrition, Huzhou Universityy, Huzhou, China; ^4^ Laboratory of Sustainable Aquaculture, International Institute of Aquaculture and Aquatic Sciences, Universiti Putra Malaysia, Port Dickson, Negeri Sembilan, Malaysia

**Keywords:** coated methionine, growth performance, digestive enzyme activity, TOR signaling pathway, gibel carp

## Abstract

This study explored the impacts of supplementation of different levels of coated methionine (Met) in a high-plant protein diet on growth, blood biochemistry, antioxidant capacity, digestive enzymes activity and expression of genes related to TOR signaling pathway in gibel carp (*Carassius auratus* gibeilo). A high-plant protein diet was formulated and used as a basal diet and supplemented with five different levels of coated Met at 0.15, 0.30, 0.45, 0.60 and 0.75%, corresponding to final analyzed Met levels of 0.34, 0.49, 0.64, 0.76, 0.92 and 1.06%. Three replicate groups of fish (initial mean weight, 11.37 ± 0.02 g) (20 fish per replicate) were fed the test diets over a 10-week feeding period. The results indicated that with the increase of coated Met level, the final weight, weight gain (WG) and specific growth rate initially boosted and then suppressed, peaking at 0.76% Met level (*P*< 0.05). Increasing dietary Met level led to significantly increased muscle crude protein content (*P*< 0.05) and reduced serum alanine aminotransferase activity (*P*< 0.05). Using appropriate dietary Met level led to reduced malondialdehyde concentration in hepatopancreas (*P*< 0.05), improved superoxide dismutase activity (*P*< 0.05), and enhanced intestinal amylase and protease activities (*P*< 0.05). The expression levels of genes associated with muscle protein synthesis such as insulin-like growth factor-1, protein kinase B, target of rapamycin and eukaryotic initiation factor 4E binding protein-1 mRNA were significantly regulated, peaking at Met level of 0.76% (*P*< 0.05). In conclusion, supplementing optimal level of coated Met improved on fish growth, antioxidant capacity, and the expression of TOR pathway related genes in muscle. The optimal dietary Met level was determined to be 0.71% of the diet based on quadratic regression analysis of WG.

## 1 Introduction

Along with the expansion of the aquaculture industry, the demand for fishmeal as a high-quality protein source for the production of aquafeeds has also increased leading to higher feed costs (1). Furthermore, the production amount of fishmeal will barely meet the aquaculture demands. Plant proteins are more widely available and cheaper than fishmeal (2), offer a cost-effective alternative (3). However, plant proteins come with challenges such as high crude fiber content, higher anti-nutritional properties, and imbalanced amino acid composition (4). Among these, unbalanced amino acid composition in plant protein mainly refers to essential amino acid deficiency (5).

High plant protein feed often results in a lack of essential amino acids (6). Methionine, an essential amino acid, is found in relatively small amounts in plant-based proteins, including soybean meal, rapeseed meal, and cottonseed meal, making it a limiting factor in these protein sources (7). Methionine plays a role in numerous metabolic processes and is critical for the growth of aquatic animals. However, a lack of dietary methionine can result in growth retardation (8), reduce feed efficiency (9), and affect body metabolism (10) and intestinal health development (11). Therefore, supplementing diets with the sufficient amount of methionine improves growth performance as demonstrated in previous studies with largemouth bass (*Micropterus salmoides*) (12), rohu (*Labeo rohita*) (13), grass carp (*Ctenopharyngodon idella*) (14), cobia (*Rachycentron canadum*) (15). Previous studies have reported that amino acids, acting as nutritional factors, can regulate the expression of protein synthesis genes through the target of rapamycin (TOR) pathway (16, 17). Moreover, supplementation of dietary methionine could activate the TORC1/S6K1 signaling pathway, promoting muscle protein content in grass carp (18). Previous study also demonstrated that dietary methionine levels protein turnover and gene expression of growth hormone (GH) and insulin-like growth factor-I (IGF-I), thereby regulating protein metabolism (19). However, the growth and metabolism of GH-IGF-modified organisms are primarily dependent on the phosphatidylinositol 3-kinase (PI3K)/protein kinase B (AKT) pathway (20). Through the PI3K/AKT signaling pathway to activate the TOR, TOR can affect protein synthesis by activating downstream S6 kinase 1 (S6K1) and 4E binding protein-1 (4E-BP1) (14).

Crystalline methionine is commonly used as a feed additive but its utilization varies across different fish species (21). Some studies have shown that crystalline amino acids have a high dissolution rate in water leading to unsynchronized protein absorption in feed (22). As a result, the addition of crystal amino acids to gastritis fish, such as crucian carp (*Carassius auratus gibelio*) (23) and mirror carp (*Cyprinus carpio*) (24), rendering them ineffective. Nevertheless, coating amino acids as opposed to crystalline methionine, has been shown to improve the utilization of amino acids by fish (25). This enhancement in amino acid utilization through coating compared to their crystalline counterparts has been substantiated by research on tilapia (*Oreochromis mossambicus*) (26) and mirror carp (27).

*Carassius auratus* gibelio has a strong adaptive ability and is an omnivorous aquaculture species, which is widely cultured crucian carp throughout China. Researchers have studied the effects of adding crystalline methionine to the feed on different sizes of gibel carp and the methionine requirement of gibel carp ranges from 0.69% to 0.98% (28–30). Thus, this experiment mainly explored the effects of different levels of coated methionine in high plant protein diets on the growth performance, serum biochemistry, liver antioxidant, intestinal digestive enzymes and expression of genes related to TOR pathway, and the optimum addition amount of coated methionine was determined by the growth parameter of gibel carp, which provided some reference for practical production.

## 2 Materials and methods

### 2.1 Experimental diets

The primary protein sources in the experimental diets included soybean meal, rapeseed meal, cottonseed protein, and fish meal, while the main fat sources were fish oil, soybean oil, and soy phospholipid. Six levels of coated methionine (0, 0.15, 0.30, 0.45, 0.60 and 0.75%) were supplemented in the diet, resulting in the final measured methionine content of 0.34 (control dietary treatment), 0.49, 0.64, 0.76, 0.92 and 1.06%, respectively (**Table 1**). The methionine levels were quantified using High-Performance Liquid Chromatography (HPLC) following a specific protocol (27).

**Table 1:** Feed composition and nutrient level of experimental diets (%, dry matter basis).

Items	Dietary Met level (%)					
	0.34	0.49	0.64	0.76	0.92	1.06
Fish meal	5	5	5	5	5	5
Soybean meal	26.8	26.8	26.8	26.8	26.8	26.8
Rapeseed meal	12	12	12	12	12	12
Cottonseed protein	10	10	10	10	10	10
Wheat meal	30.1	30.1	30.1	30.1	30.1	30.1
Soybean oil	1	1	1	1	1	1
Soy lecithin	1.5	1.5	1.5	1.5	1.5	1.5
Fish oil	1	1	1	1	1	1
Extruded corn	7.5	6.0	4.5	3.0	1.5	0
10% Coated methionine	0	1.5	3.0	4.5	6.0	7.5
Vitamin premix^1^	0.5	0.5	0.5	0.5	0.5	0.5
Mineral premix^2^	0.2	0.2	0.2	0.2	0.2	0.2
Sodium carboxymethyl cellulose	2	2	2	2	2	2
Choline chloride	0.2	0.2	0.2	0.2	0.2	0.2
Ca(H_2_PO_4_)_2_	2	2	2	2	2	2
MgSO_4_	0.2	0.2	0.2	0.2	0.2	0.2
Nutrient levels (%)						
Crude protein	31.82	31.83	31.94	32.05	32.01	31.97
Crude lipid	5.12	5.23	5.36	5.32	5.12	5.10
Ash	6.54	6.52	6.50	6.47	6.45	6.43
Methionine	0.34	0.49	0.64	0.76	0.92	1.06

1 The premix provided the following per kg of diets: VA 8000IU, VC 500mg, VD 3000IU, VE 60mg, VK 35mg, VB_1_ 15mg, VB_2_ 30mg, VB_6_ 15mg, VB_12_ 0.5mg .

2 The premix provided the following per kg of diets: FeSO_4_·7H_2_O 754.56 mg, CuSO_4_·5H_2_O 23.81 mg, MnSO_4_·H_2_O 168.29 mg, ZnSO_4_·7H_2_O 440.00 mg, Na_2_SeO_3_ 2.26 mg, KI 0.79 mg, CoCl_2_ 2.21 mg, Zeolite meal 604.08mg.

The feed ingredients used in the experiment were pulverized and passed through a 60 mesh screen. All the ingredients were mixed thoroughly according to the formulation (**Table 1**) and the mix blends were preconditioned with water prior to extrusion to produce pellets with a 2.5 mm diameter. Then the pellets were oven dried at 35°C overnight and stored in a refrigerator at -20°C.

### 2.2 Experimental fish and rearing

The 10-week feeding trial was conducted at the College of Life Sciences, Huzhou University. Prior to the feeding trial, all the fish were acclimatized for 2 weeks and after acclimatization, a total of 360 healthy gibel carp of similar sizes were selected for the study. The fish (initial, 11.37 ± 0.02 g) were randomly divided into 6 dietary treatments, each with 3 replicates and 20 fish per tank. The experimental recirculating system was housed outdoors and the fish were fed manually twice a day (8:30 am and 17:30 pm) at 2.5% to 3% of body weight. Additionally, feed residues were collected from the culture system 30 min after feeding. During the feeding trial, the water conditions were consistently within the specified ranges, with the water temperature ranging from 20°C to 32°C, dissolved oxygen levels maintained at or above 6.0 mg/L, ammonia nitrogen levels kept below 0.3 mg/L, and nitrite levels maintained below 0.05 mg/L.

The weight and number of fish in each tank were recorded weekly. At the end of the feeding trial, weights of the fish, viscera, and hepatopancreas were recorded, and the full length and body length of the fish were measured. The calculation formula is as follows:


Survival rate (%) = 100× final fish number/initial fish number;

$$\begin{array}{c} \text{Weight gain }\left(\%\right)\;=\;100\times \;[\left(\text{final body weight}-\text{initial body weight}\right)\\ /\text{initial body weight}]; \end{array}$$

$$\begin{array}{c} \text{Specific growth rate }\left(\%/\text{day}\right)\;=100\times \;[(\text{ln final body weight}\\ -\text{ln initial body weight})/\text{days}]; \end{array}$$

$$\begin{array}{c} \text{Feed intake rate }\left(\%/\text{day}\right)\;=100\times \text{dry feed intake}/\\ \;\left[\text{days}\times \left(\text{initial body weight}+\text{final body weight}\right)/2\right]; \end{array}$$

Feed conversion rate = 100×dry feed fed/weight gain;

Viscerosomatic index (%) = 100×viscerosomatic weight/final body weight;

Hepatosomatic index (%) = 100×hepatosomatic weight/final body weight;

$$\text{Condition factor }\left(\%\right)\;=\;100\times \text{final body weight}/{\left(\text{final body length}\right)}^{3}.$$

### 2.3 Sample collection

At the end of the feeding trial, 6 gibel carp were chosen at random from each tank and anesthetized using MS-222 (at a concentration of 400 mg/L). Blood was attained via the tail vein and centrifuged (3500 r/min, 10 min) at 4°C to obtain plasma serum, which was stored in a refrigerator at -80°C. Subsequently, samples of hepatopancreas, intestine and muscle were collected. The samples were placed in centrifuge tubes, swiftly transferred into liquid nitrogen, and subsequently stored in a -80°C refrigerator.

### 2.4 Muscle composition analysis

A proximate composition analysis was performed following the standard protocol (31). Moisture content was measured through oven drying at 105°C using a DHG-9240A oven (Shanghai Jinghong Experimental Equipment Co., Ltd., China), while ash content was assessed with a muffle furnace set at 550°C (SX2-8-10, Shanghai Yiheng Technology Co., Ltd., China). The determination of crude protein content utilized the Dumas combustion method, employing an Elementar rapid N exceed apparatus (Elementar Trading (Shanghai) Co., Ltd., China), and the crude fat content was analyzed using the Soxhlet extraction method.

### 2.5 Biochemical assays

Saline was mixed with liver and intestine tissues in a 1: 9 (w: v) ratio and homogenized. The homogeneity was then centrifuged at 4°C at 3500 rpm for 10 minutes, and the supernatant was collected for subsequent assays. Serum biochemical assays were conducted for aspartate aminotransferase (AST, Cat. No. C010-1-1) and alanine aminotransferase (ALT, Cat. No. C009-1-1). Additionally, antioxidant enzyme activities, including catalase (CAT, Cat. No. A007-1-1), superoxide dismutase (SOD, Cat. No. A001-1), and lipid peroxidation marker malondialdehyde (MDA, Cat. No. A003-1), were measured in the hepatopancreas. Enzymatic activities of lipase (Cat. No. A054-1-1) and amylase (Cat. No. C016-1-1) were determined in the intestine. All assay kits were sourced from Nanjing Jiancheng Bioengineering Institute. The activity of intestinal protease was assessed using the folin-phenol reagent method (32).

### 2.6 Gene expression analysis

Total RNA was extracted from the muscle using a kit for the rapid extraction of RNA (RN28-EASYspin). The concentration and purity of total RNA were determined by spectrophotometry (A260: A280 nm), with reference to the ratio specified in the kit instructions (1.8-2.2). Subsequently, the extracted mRNA was synthesized into cDNA using a reverse transcription kit (MonScript™RTIII All-in-One Mix).

Primers were designed using online Primer through NCBI website. The determination of real-time PCR, the CFX-96 system was used (BioRad Laboratories, Inc., USA). Real-time PCR analysis of mRNA levels was performed according to the kit instructions (Mon Amp™SYBR^®^Green qPCR Mix). The total reaction system was 20 µL, containing diluted cDNA 1 µL, reverse and forward primers 0.4 µL (10µM), nuclease-free water 8.2 µL, and monamp™SYBR^®^Green qPCR Mix 10 µL. PCR conditions refer to Luo et al. (33). The mRNA relative expression of target gene was calculated by 2^-ΔΔCt^ method, and β-actin gene was a housekeeping gene. **Table 2** is the primers for fluorescence quantitative analysis.

**Table 2:** Primer sequences for RT-PCR in the experiment.

Target genes	Primer sequence (5’-3’)	Gene ID
β-action	AGTACGATGAGTCTGGCCCT ATCCTGAGTCAATGCGCCAA	AB039726
			PI3K	TTGACACAGAAAGGGGTCCG CTTCCAGGAGCGTTCGTCAT	XM_052557910.1
AKT	GCGGGAGGCTGGATACTAAC GTGGCATCCGAAGAATTCGC	XM_052562157.1
TOR	GCACAAATTGATGGCACGGT GCAGCACTGCCTCAAAGTTC	KF772613.1
4E-BP1	CAATTCCCACCACCAGACGA CCAACGGAGAGCTACGACAG	XM_052536897
S6K1	CCAGCCGGAGGAAAATGTCT GCGTGCCGTTCACTTCAAAA	EF373665.1
IGF-1	AGGGGATGTCTAGCGGTCAT AGAGACAGCGCATGGTACAC	KF813006.1

PI3K, Phosphatidylinositol 3-kinase; Akt, Protein kinase B; TOR, Target of rapamycin; 4E-BP1, Eukaryotic initiation factor 4E binding protein-1; S6K1, Ribosomal protein S6 kinase 1; IGF-1, Insulin-like growth factor-1.

### 2.7 Data analysis

All data were analyzed using SPSS 25.0 software and expressed as mean (n=3) ± standard error of the mean (SEM). Each variable evaluated was analyzed using analysis of variance (ANOVA), and data from each dietary treatment were compared using Duncan’s method. A statistically significant difference was indicated when *P*< 0.05. Comparisons were made using orthogonal polynomials to assess the significance of linear and quadratic models for all dependent variables at the level of the coated methionine.

## 3 Results

### 3.1 Growth performance

At the conclusion of 10-week feeding trial, the SR of gibel carp was 100% (**Table 3**). As dietary levels of coated methionine (Met) increased, final body weight, weight gain (WG), and specific growth rate initially increased, peaking at 0.76% dietary treatment, before declining (*P*< 0.05). Conversely, feed conversion ratio (FCR) exhibited an opposite trend, recording its lowest value in the 0.76% dietary treatment, though this difference was not statistically significant (*P*> 0.05). Feed intake rate, FCR, viscerosomatic index, hepatosomatic index, and condition factor remained unaffected by the Met levels in the diets (*P*> 0.05).

**Table 3:** Effects of dietary coated Met on growth performance of gibel carp.

Items	Dietary Met levels (%)						SEM	*P* values	Polynomial contrasts	
	0.34	0.49	0.64	0.76	0.92	1.06			Linear	Quadratic
IBW (g)	11.25	11.27	11.26	11.26	11.27	11.26	0.004	0.884	0.671	0.724
FBW (g)	23.25^b^	23.80^ab^	23.98^ab^	24.32^a^	23.87^ab^	23.27^b^	0.119	0.031	0.822	0.002
SR (%)	100	100	100	100	100	100	–	–	–	–
WG (%)	106.61^b^	111.26^ab^	112.98^ab^	115.88^a^	111.82^ab^	106.63^b^	1.049	0.034	0.848	0.002
SGR (%/d)	1.04^b^	1.07^ab^	1.08^ab^	1.10^a^	1.07^ab^	1.04^b^	0.007	0.033	0.841	0.003
FIR (%/day)	2.34	2.34	2.34	2.34	2.34	2.35	0.005	0.993	0.796	0.853
FCR	2.36	2.29	2.27	2.24	2.29	2.37	0.018	0.212	0.971	0.023
VSI (%)	9.89	9.92	10.30	10.15	10.15	10.10	0.084	0.754	0.353	0.400
HSI (%)	4.28	4.52	4.76	4.64	4.57	4.86	0.101	0.662	0.151	0.325
CF (g/cm^3^)	2.84	2.91	2.88	2.97	2.80	2.76	0.026	0.199	0.223	0.069

SEM, Standard error of the mean. There are significant differences between different letters (P< 0.05). IBW, Initial body weight; FBW, Final body weight; SR, Survival rate; WG, Weight gain; SGR, Specific growth rate; FIR, Feed intake rate; FCR, Feed conversion rate; VSI, Viscerosomatic index; HSI, Hepatosomatic index; CF, Condition factor.

**Figure 1:**
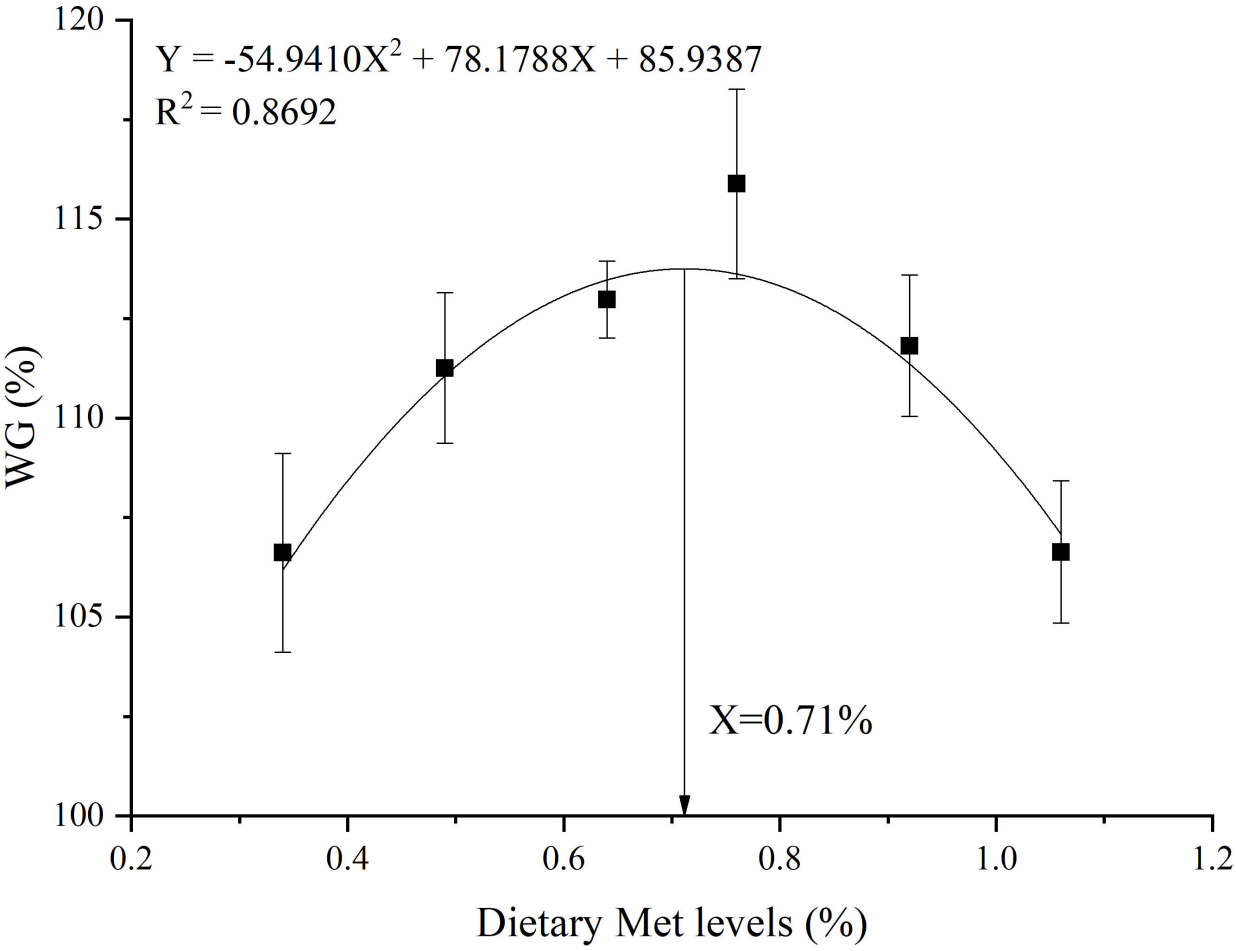
Quadratic regression analysis of WG against varying dietary coated Met levels.

Quadratic regression analysis showed that the WG was the highest at 0.71% Met level. Moreover, 0.34% was derived from the basal diet, and 0.37% was derived from the coated Met (**Figure 1**).

### 3.2 Muscle composition

Dietary coated Met significantly increased muscle crude protein content, which peaked at 0.64% dietary treatment and then stabilized (**Table 4**). Dietary coated Met did not show significant effects on muscle moisture, crude fat and ash content (*P*> 0.05).

**Table 4:** Effects of dietary coated Met on muscle composition of gibel carp.

Items	Dietary Met levels (%)						SEM	*P* values	Polynomial contrasts	
	0.34	0.49	0.64	0.76	0.92	1.06			Linear	Quadratic
Moisture	77.56	76.99	76.97	77.25	77.39	77.15	0.085	0.300	0.744	0.468
Crude protein	17.34^b^	17.60^ab^	17.79^a^	17.79^a^	17.79^a^	17.76^a^	0.053	0.049	0.009	0.002
Crude lipid	1.25	1.08	1.07	1.14	0.97	0.81	0.051	0.193	0.012	0.040
Ash	1.54	1.53	1.53	1.55	1.50	1.46	0.019	0.853	0.249	0.411

SEM, Standard error of the mean. There are significant differences between different letters (P< 0.05).

### 3.3 Serum biochemical parameters

Serum ALT activity decreased significantly with increasing levels of coated Met, reaching its lowest level in the 1.06% dietary treatment (**Table 5**). AST activity was not markedly influenced by the level of coated Met (*P*> 0.05). However, AST activity showed a linearly decrease by polynomial analysis (*P*< 0.05).

**Table 5:** Effects of coated Met on serum biochemical indices of gibel carp.

Items	Dietary Met levels (%)						SEM	*P* values	Polynomial contrasts	
	0.34	0.49	0.64	0.76	0.92	1.06			Linear	Quadratic
AST (U/L)	45.63	43.21	44.21	43.27	40.98	36.98	0.991	0.155	0.010	0.023
ALT (U/L)	8.15^a^	7.91^ab^	5.24^bcd^	6.49^abc^	4.94^cd^	3.55^d^	0.437	0.006	0.000	0.001

SEM, Standard error of the mean. There are significant differences between different letters (P< 0.05). AST, Aspartate aminotransferase; ALT, Alanine aminotransferase.

### 3.4 Hepatopancreas antioxidant capacity

Hepatopancreas MDA content (**Table 6**) generally decreased and then slightly increased with the level of coated Met, reaching the lowest level in the 0.92% dietary treatment (*P*< 0.05), while the SOD activity generally showed the opposite trend, peaking in the 0.64% dietary treatment (*P*< 0.05). However, CAT activity was not significantly different among the dietary treatments (*P*> 0.05).

**Table 6:** Effects of dietary coated Met on liver antioxidant of gibel carp.

Items	Dietary Met levels (%)						PSE	*P* values	Polynomial contrasts	
	0.34	0.49	0.64	0.76	0.92	1.06			Linear	Quadratic
CAT (U/mg prot)	40.93	46.89	43.68	48.34	46.15	49.55	1.465	0.595	0.664	0.411
MDA (nmol/mg prot)	3.47^ab^	4.44^a^	2.50^bc^	2.66^bc^	2.03^c^	2.68^bc^	0.188	0.001	0.188	0.231
SOD (U/mg prot)	134.05^c^	151.25^ab^	163.98^a^	152.21^ab^	151.64^ab^	144.84^bc^	2.634	0.009	0.413	0.003

SEM, Standard error of the mean. There are significant differences between different letters (P< 0.05). CAT, Catalase; MDA, Malondialdehyde; SOD, Superoxide dismutase.

### 3.5 Intestinal digestive enzyme parameters

Intestinal amylase and protease activities (**Table 7**) were higher and then lower with increasing levels of coated Met, reached a high level in the 0.92% dietary treatment (*P*< 0.05). Lipase activity tended to increase, but there was no significant effect by different levels of coated Met (*P*> 0.05).

**Table 7:** Effects of dietary coated Met on intestinal digestive enzyme activities of gibel carp.

Items	Dietary Met levels (%)						SEM	*P* values	Polynomial contrasts	
	0.34	0.49	0.64	0.76	0.92	1.06			Linear	Quadratic
Amylase (U/mg prot)	62.69^c^	64.08^bc^	70.07^ab^	74.56^a^	74.75^a^	66.70^bc^	1.377	0.008	0.264	0.048
Lipase (U/g prot)	12.71	15.18	20.00	20.25	17.91	16.83	1.020	0.242	0.172	0.038
Proteinase(U/mg prot)	5.48^c^	8.74^b^	9.71^ab^	10.56^ab^	11.65^a^	11.13^ab^	0.570	0.002	0.006	0.002

SEM, Standard error of the mean. There are significant differences between different letters (P< 0.05).

### 3.6 TOR pathway gene expression

**Figure 2:**
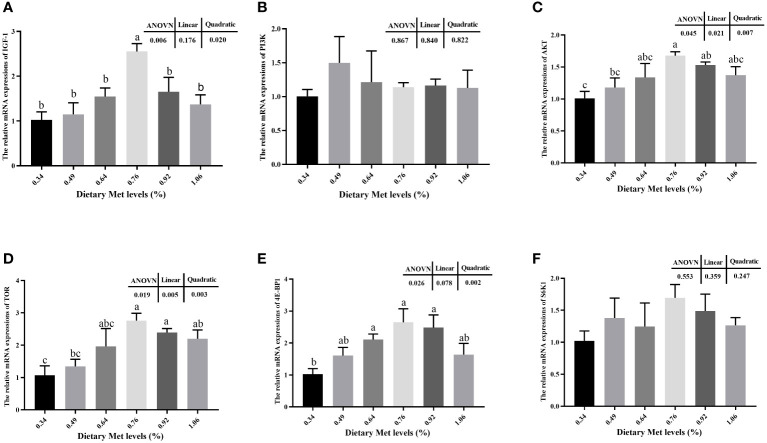
Effects of dietary coated Met on the expression of genes related to TOR signaling pathway. There are significant differences between different letters (*P<* 0.05). IGF-1, Insulin-like growth factor-1; PI3K, Phosphatidylinositol 3-kinase; Akt, Protein kinase B; TOR, Target of rapamycin; 4E-BP1, Eukaryotic initiation factor 4E binding protein-1; S6K1, Ribosomal protein S6 kinase 1. **(A)** The relative mRNA expressions of IGF-1. **(B)** The relative mRNA expressions of PI3K. **(C)** The relative mRNA expressions of AKT. **(D)** The relative mRNA expressions of TOR. **(E)** The relative mRNA expressions of 4E-BP1. **(F)** The relative mRNA expressions of S6K1.

The addition of coated Met to the feed significantly increased the muscle mRNA expression levels of IGF-1, AKT, TOR, and 4E-BP1 (*P<* 0.05), and all peaked at 0.76% dietary treatment (**Figure 2**). Moreover, the mRNA expression levels of PI3K and S6K1 tended to increase but were not significantly dietary treatment (*P>* 0.05).

## 4 Discussion

### 4.1 Effects of coated methionine on growth performance of gibel carp

Weight gain (WG) is an important indicator of normal growth of aquatic animals at a given growth stage (34). During the 70-day feeding period, the WG results for the fish ranged from 106.61% to 115.88%. The results showed normal growth, aligning with findings from other reports. For instance, after 60 days of feeding, gibel carp that started at 13.20 g showed WG rates ranging from 88.44% to 106.99% (35). Similarly, gibel carp initiated the trial with a baseline weight of 28.00 g demonstrated WG percentages ranging from 74.19% to 93.60% over the corresponding 60-day period (36).

Methionine (Met) plays an important role in fish growth and is an essential amino acid. Based on previous studies, inadequate dietary intake of Met can reduce WG in turbot (*Scophthalmus maximus*) (37) and Atlantic salmon (*Salmo salar*) (8). The excessive Met in the feed also leads to a decrease in the WG of yellow catfish (*Pelteobagrus fulvidraco*) (38). This experiment showed that as the level of coated Met in the diet increased, the WG and SGR of gibel carp initially improved, then diminished, reaching a peak at the 0.76% dietary treatment level. The results demonstrate that both an overabundance and a deficiency of Met in the feed adversely affect growth, whereas optimal supplementation of Met enhances growth. This trend has been reported in other studies for mirror carp (27) and cobia (22). In the current study, the addition of coated Met did not lead to significant differences in FCR among dietary treatments, which is consistent with findings in turbot (39). Studies on grouper (*Epinephelus coioides*) have shown that the supplementation of Met to the diet causes differences in CF among dietary treatments (40). In this study, coated Met levels did not significantly change HSI, VSI and CF. However, the HSI index of gibel carp in this experiment was higher than that reported by Jia et al. (41), but lower than the values reported by Wang et al. (29). This difference may be attributed to variations in feed composition and fish size of gibel carp.

Previous studies on this species (51.0 g), DL-Met was added to the feed (0.44% Met in basic feed), and the optimum levels of Met were 0.73% and 0.98%, respectively (29). Another study showed that adding DL-Met to the diet, resulted in an optimal Met level of 0.89% for gibel carp (with the basal diet containing 0.57% Met) (41). In this study, the optimal dietary Met content for gibel carp was determined to be 0.71% through quadratic regression analysis, with the basal diet containing 0.34% Met and an additional 0.37% derived from coated Met. The results were consistent with the previously reported dietary Met levels of 0.71% and 0.73% (29, 30), but slightly less than 0.89% (41). This discrepancy may be attributed to variations in feed nutrient composition and types of Met used. Nevertheless, it is important to note that the present study context differs from previous studies in the natural basal diet’s Met content. Specifically, previous research reported higher basal Met levels, around 0.44%, whereas our study utilized a basal diet with a lower Met content of 0.34% due to formulation with a high plant-based protein diet.

### 4.2 Effect of coated methionine on serum biochemical indexes of gibel carp

As an index of fish physiological state, serum biochemical index can reflect the health status of the body (42). AST and ALT are two key enzymes involved in the normal metabolism of amino acids (43). When hepatic metabolism is disturbed or impaired, it will lead to excessive increase of AST and ALT in serum (44). Previous studies on gibel carp (30) and Jian carp (*Cyprinus carpiovar.* Jian) (45) have found that the deficiency or excess of Met in feed can lead to an increase in serum transaminase activity, which negatively affects liver function in fish. The experimental results showed that AST in serum decreased with the addition of coated Met in the diet, but did not cause differences among dietary treatments, which was similar to the experimental results of golden pomfret (*Trachinotus ovatus*) (46). Similarly, ALT showed a downward trend after the addition of coated Met. AST and ALT activities were higher in the Met-deficient group, suggesting that the deficiency of Met in the diet may have disrupted the normal metabolism of the liver, leading to impaired liver function. The addition of coated Met could reduce serum ALT and AST activities, probably due to the interaction between Met and other amino acids, which maintains normal protein metabolism in the liver and is beneficial to liver health (47).

### 4.3 Effect of coated methionine on antioxidant capacity of liver in gibel carp

The liver of fish is the main immune organ and metabolic site, and the physiological status of the liver affects the health of the body (48). Superoxide dismutase and catalase are two enzymes that can scavenge free radicals in the body and are closely related to the antioxidant capacity of the body (49). MDA, as a lipid oxidation product, directly reflects the degree of lipid oxidation in an organism and serves as an indicator of its physiological state (50). In a report on hybrid striped bass (*Morone chrysops×M. siaxatilis*), it was found that the shortage of Met in the diet may cause changes in the body’s antioxidant capacity, leading to oxidative stress (51). Previous study reported that appropriate Met level could increase the antioxidant capacity and ameliorate oxidative damage in grouper (52). Met is an important part of the cellular antioxidant system and protects cells from oxidative damage (53). In this study, gibel carp fed diets supplemented with coated Met showed lower MDA content and higher SOD activity compared to Met-deficient dietary treatments. These results indicated that Met deficiency can cause an increase in MDA content in the liver of gibel carp, which may cause oxidative damage to the body (54). However, the addition of properly coated Met decreased the MDA content in the liver and increased the activity of SOD in the body, which was similar to the results of grouper (52) and hybrid striped bass (51).

### 4.4 Effect of coated methionine on intestinal digestive enzymes of gibel carp

The intestinal functions of digestion and absorption are essential for the appropriate uptake of nutrients and growth in fish (55). Efficient digestion and absorption are necessary for optimal fish growth. Protease, amylase, and lipase are key enzymes involved in digestion (56). The digestive and absorptive capacity of the fish is reflected by the enzyme activity in the intestine (57). Increasing digestive enzyme activity promotes nutrient assimilation, leading to better growth performance (58). Previous studies on grass carp reveal that adequate dietary Met level can notably elevate the intestinal digestive enzyme activity (11). Studies have shown that Met deficiency may result in decreased activity of digestion enzymes, and that dietary supplementation with coated Met may markedly increase intestinal digestion enzyme activity. Meanwhile, the increase of intestinal digestive enzyme activity was similar to the trend of WG. We speculate that this is due to an improvement in the activity of digestive enzymes in the gut, which improves the digestion of nutrients and thus promotes the growth of gibel carp. This was confirmed in another study of mirror carp, and the addition of Met promoted intestinal villus height and muscle thickness, which had a positive effect on intestinal development (34).

### 4.5 Effect of coated methionine on the expression of TOR pathway related genes in gibel carp

In fish, IGF-1 plays a crucial role in growth promoting, bone formation and metabolic regulation (59). The present study elucidated that including coated Met in the diet up-regulates mRNA expression levels of IGF-1 gene. Moreover, the gene expression level trend upward was the equivalent of WG. Its hypothesized that dietary supplementation with coated Met could promote fish growth and development by up regulating IGF-1 gene expression, and similar results were found in hybrid grouper (60) and rainbow trout (*Oncorhynchus mykiss*) (61). Previous study have reported that binding of IGF-1 to receptors on the cell membrane in muscle cells activates downstream TOR via the PI3K and AKT signaling pathways, and that TOR promotes intracellular protein synthesis through activation of downstream 4E-BP1 and S6K1 (62). The present study illustrated that increased of dietary coated Met markedly influenced the expression of AKT, TOR, and 4E-BP1 gene mRNAs in the TOR pathway. Simultaneously, it was noted in the present study that there was a comparable trend in the expression of genes linked to the TOR pathway, corresponding to increased CP content in muscle (14). Therefore, a hypothesis was raised suggesting Met, as a precursor of protein synthesis, activates the expression of TOR pathway-related genes and promotes protein synthesis in muscle, thus affecting the protein deposition and growth of fish. Similar results have been observed in rainbow trout (19), cobia (7) and grass carp (18).

## 5 Conclusions

In conclusion, the addition of optimal level of coated Met significantly increased the WG and SGR of gible carp. Furthermore, it enhanced the antioxidant capacity of the hepatopancreas, improved the activity of intestinal digestive enzymes, and up regulated the expression of genes associated with protein synthesis in muscle. The optimal dietary methionine level, as determined through quadratic regression analysis of WG, is recommended to be 0.71%.

## Data availability statement

The original contributions presented in the study are included in the article/supplementary material. Further inquiries can be directed to the corresponding author.

## Ethics statement

The animal study was approved by The Animal Experimental Ethics Committee of Huzhou University (20180306). The study was conducted in accordance with the local legislation and institutional requirements.

## Author contributions

YD: Formal analysis, Methodology, Writing – original draft, Writing – review & editing. XL: Formal analysis, Writing – original draft. XS: Formal analysis, Methodology, Writing – review & editing. JZ: Methodology, Writing – review & editing. HX: Methodology, Writing – review & editing. CC: Validation, Writing – review & editing. QX: Methodology, Resources, Supervision, Validation, Writing – review & editing.
